# Xpert MTB/RIF assay did not improve diagnosis of pulmonary tuberculosis among child contacts in Rwanda

**DOI:** 10.11604/pamj.2018.30.39.12600

**Published:** 2018-05-17

**Authors:** Francine Mwayuma Birungi, Brian van Wyk, Jeannine Uwimana, Joseph Ntaganira, Stephen Michael Graham

**Affiliations:** 1 Department of Epidemiology and Biostatistics, School of Public Health of the College of Medicine and Health Sciences, University of Rwanda, Kigali, Rwanda; 2 Faculty of Community and Health Sciences, University of Western Cape, Cape Town, South Africa; 3 Centre for International Child Health, University of Melbourne Department of Paediatrics and Murdoch Children’s, Research Institute, Royal Children’s Hospital, Melbourne, Australia; 4 International Union Against Tuberculosis and Lung Disease, Paris, France

**Keywords:** Pulmonary tuberculosis, child, contact screening, Xpert MTB/RIF assay, gastric lavage

## Abstract

**Introduction:**

To report on the diagnostic yield using the Xpert MTB/RIF assay on gastric lavage samples from children (<15 years) who were household contacts of tuberculosis (TB) cases in Kigali, Rwanda.

**Methods:**

A cross-sectional study was conducted among 216 child contacts of index cases with sputum smear-positive TB over a 7 month period, from 1^st^ August 2015 to 29^th^ February 2016. Child contacts with tuberculosis-related symptoms or abnormal chest X-ray had sputum collected by gastric lavage on two consecutive days and samples were examined by smear microscopy, Xpert MTB/RIF assay and solid culture.

**Results:**

Of the 216 child contacts, 94 (44%) were less than 5 years of age. Most of them 84 (89%) were receiving isoniazid preventive therapy at the time of screening. Thirty seven out of 216 children had TB-related symptoms. Only 4 (10.8%) were clinically diagnosed with TB; and none had bacteriologically confirmed tuberculosis.

**Conclusion:**

The use of Xpert MTB/RIF assay did not contribute to bacteriological confirmation of active TB in child contacts in this study. The low prevalence of tuberculosis in child contacts in this study may reflect the high coverage of preventive therapy in young (<5 years) child contacts. The low sensitivity of Xpert MTB/RIF assay in contacts may also suggest likely reflection of paucibacillary disease.

## Introduction

Tuberculosis (TB) is a major cause of morbidity and mortality among children (0-14 years) in resource-limited countries [1]. The World Health Organisation (WHO) estimated that 10% of the 9 million TB incident cases occurred in children in 2015 and that there were 210,000 TB-related deaths in children, including 170,000 in Human Immunodeficiency Virus (HIV)-uninfected children [2]. The annual report of Rwanda's 2013-2014 National Tuberculosis Program (NTP) indicated that child TB cases represented 6% of all notified TB cases, below the national target of 12% [3]. Among these cases, 68% were pulmonary TB and 22% were bacteriologically confirmed. These data suggest under-detection of TB in children in Rwanda, especially of clinically diagnosed cases. There are well recognised challenges with detection and diagnosis, particularly in young children (<5 years) with paucibacillary disease, difficulty in obtaining samples and clinical overlap of TB with other common diseases such as severe pneumonia and malnutrition [4-7]. Young children (< 5 years) who develop active TB subsequent to infection with Mycobacterium tuberculosis, usually do so within one year of infection [8]. Children who are close to a TB index case are at high risk of TB infection [9-12]. Without any intervention, 5-10% of infected children will develop active TB within one year, with the highest prevalence of TB at the time of screening being in young children (< 5 years) [8,13]. Screening of child contacts of TB cases, prioritising index cases with sputum smear-positive pulmonary TB, is almost universally recommended and plays two important roles which include identification and evaluation of symptomatic contacts of any age requiring further diagnostic assessment of TB for early treatment (i.e. active case finding), and the provision of preventive therapy to “high-risk” contacts that do not have active TB [14]. Since 2006, WHO has recommended a symptom-based screening approach that allows the initiation of contact management and the provision of preventive therapy for asymptomatic young child contacts at the household or primary care level [15,16]. However, symptomatic contacts need further evaluation for TB and this remains challenging at the primary or secondary care level given the widely recognized limitations of current diagnostic tools especially in young children.

In 2013, the WHO endorsed the Xpert MTB/RIF assay for use in children [15,17]. The Xpert MTB/RIF assay offers advantages over smear microscopy for acid-fast bacilli. Research studies reported Xpert MTB/RIF assay to be three times more sensitive than sputum smear but with sensitivity compared to culture lower in outpatient children than in inpatients (48% versus 70%) [18]. Under programmatic conditions in a large study in India, Xpert MTB/RIF assay had twice the yield of smear with similar yield from sputum collected by gastric aspirate or induced sputum [19]. The Xpert MTB/RIF assay can be implemented in a peripheral laboratory with a result in less than two hours that includes information on rifampicin resistance [17,20]. Hence, the Xpert/MTB/RIF assay can potentially improve case detection among child contacts compared to smear while overcoming other constraints to active screening that include reducing the time, cost and complexity to the individual, family and health service incurred by the need for multiple visits to a hospital to complete evaluation for TB [21,22]. Few studies have evaluated the performance of the Xpert MTB/RIF assay in the context of contact screening in children where the children with TB are outpatients and likely to have early disease that is paucibacillary compared to hospital-based studies of more advanced cases passively detected [18,23,24]. Further, no previous studies have utilised gastric lavage (GL) in the evaluation of symptomatic child contacts. The Xpert MTB/RIF assay was introduced as a diagnostic tool for all children suspected of having TB in Rwanda in 2014. However, only samples from self-expectorated sputum have been used. This study aims to evaluate the diagnostic performance of the Xpert MTB/RIF assay in sputum collected by GL in symptomatic children who are contacts of index cases with sputum smear-positive TB.

## Methods

**Study design and setting**: This is a cross-sectional study of child contacts of sputum smear-positive index cases who were detected between 1^st^ August 2015 and 29^th^ February 2016 at 13 primary health centres (PHCs) based in Kigali, the capital city of Rwanda. Kigali reports the highest prevalence of TB in Rwanda and around 30% of Rwanda's total pulmonary TB (PTB) cases [**25**]. Kigali city has four referral hospitals, four District hospitals which are all TB diagnostic and treatment centres and 35 PHCs. Of the PHCs, 23 provide TB diagnostic and treatment services; thus, potential entry points for TB cases. A PHC was selected for inclusion in this study if it reported an average of at least 10 sputum smear-positive PTB cases during the first half (January to June) of 2015.

**Study population**: Index cases diagnosed with sputum smear-positive PTB between August 2015 and February 2016 who had at least one child less than 15 years old, but who were not a member of a household to which a previous selected index case belonged and still living in Kigali city, were eligible for inclusion in the study. Identified index cases were requested, either via telephone calls or through trained community health workers (CHWs), to bring children with whom they live with to their PHC on a specific day to coincide with visits to that PHC by data enumerators. Child contacts were defined as those contacts of less than 15 years who shared the same household with a selected index case within the 3 months prior to the diagnosis of the index case. Therefore, all child contacts born after the index cases had started treatment or were not leaving with the index cases prior to the diagnosis of the index cases were excluded. Eligible children were enrolled following written informed consent by the parent or caregiver and children of 7 years and older also signed an assent form.

**Data collection and management**: A structured questionnaire adapted from screening guidelines [26,27] was pre-tested and modified during a pilot study in two selected sites. Twelve data enumerators were trained to conduct interviews with parents/caregivers of selected child contacts and to collect data from TB registers and index case folders, using standardised data collection forms. We also trained 20 CHWs to explain the study to the parents/caregivers, and sensitise them to bring child contacts for screening at the PHCs. Data of the index case included: result of smear microscopy, demographic data, address of residence and telephone number. The uptake of IPT among child contacts following diagnosis of the index case was also recorded. The recorded data were validated by the index case, parents or caregivers of selected children once they were identified in order to ensure the accuracy of the data. The demographics and medical history of index cases were recorded; and all eligible children underwent clinical screening including nutritional assessment and Chest X-ray (CXR). The clinical screening focussed on symptoms suggestive of TB: cough for ≥ 2 weeks, haemoptysis, fever, failure to gain weight, absence of appetite, fatigue, and the presence of lymphadenopathy. Anteroposterior and lateral CXR were also performed on all the 216 children; and read by two independent experienced general practitioners, trained in interpreting CXR and blinded to the clinical details of participants and proofread by an experienced radiologist. Children with symptoms suggestive of TB and/or CXR “consistent with active TB”, as described in Table 1, were given antibiotics for seven days as recommended by the current TB diagnostic algorithm in the country. Those children were thereafter reassessed. Children with persistence of symptoms despite appropriate treatment were referred to a district hospital as outpatients for sputum collection through GL. A trained nurse, under supervision of a senior paediatrician, collected a sputum sample (3-4ml), using GL technique, on two consecutive mornings from the children after six hours of fasting. The samples were directly transported to Kigali teaching hospital laboratory, a qualified high performance diagnostic mycobacteriology laboratory, where they were processed by trained technicians and investigated by smear microscopy, Xpert MTB/RIF assay and solid culture within two hours subsequent to their collection. Children diagnosed with TB were treated in accordance with the Rwanda NTP treatment guidelines [28]. Young child contacts of less than 5 years of age and with no evidence of active TB were offered IPT for 6 months as per national guidelines if they were not already receiving IPT at the time of screening.

**Table 1:** Operational definition used in this study

Symptoms suggestive of tuberculosis
Persistent unexplained fever: a one-week unexplained fever of greater than 38^o^C have been reported by parent or caregiver or at least once objectively recorded
Cough for more than 2 weeks: a story of persistent, unremitting cough for more than two weeks not responding to the standard therapy
Documented weight loss or failure to thrive: unexplained weight loss for more than 5% compared with the highest weight recorded in last 3 months
Malnourished: weight for height Z score (see definition four below)
CXR “consistent with active TB” if there is a positive response to any of the radiographic features, at the same location, by at least 2 independent radiologist reviewers
Air compression and/or tracheal displacement
Soft tissue density suggestive of lymphadenopathy
Air space opacification
Nodule picture (miliary or larger widespread) and bilateral
Pleural effusion
Cavities
Calcified parenchyma and
Vertebral spondylitis
Tuberculosis disease; if the child met the following criteria
Confirmed TB: Presence of one or more symptoms suggestive of TB, a chest radiography “consistent" with active TB and microbiological confirmation (in this study Xpert MTB/RIF assay test and/or culture positive)
Unconfirmed TB: as our study is constituted by child contacts, we will consider in this category a child who will display at least one of the symptoms suggestive of TB, CXR consistent with active TB
Unlikely TB: symptomatic child contacts suspected of TB whose symptoms and/or CXR consistent with active TB spontaneously improved after seven days of antibiotics without receiving any TB treatment
Nutritional assessment using Weight-for-Height
Normal : Z score ≥ -2 of the WHO median
Moderate malnutrition: Z score -3 to < -2 of the WHO median
Severe malnutrition: Z score < -3

Abbreviation WHO: World Health Organisation; CXR: Chest X-ray

**Laboratory procedure**: For Xpert MTB/RIF assay test, 2ml of buffer, a tampon solution of Xpert MTB/RIF assay test, was added to 1ml of fresh sample. It was then shaken and stood for 10 minutes and shaken again and stood for further 5 minutes and then, 2.5 ml of the mixed solution was transferred into the Xpert cartridge, scanned and tested. The result was read two hours later. For solid culture, 2ml of fresh sample was decontaminated with 2 ml of sodium hydroxide and then the mixed solution was neutralized with hydrochloric acid before centrifuging at 3000xg for 15 minutes by using aerosol free centrifuge cups. The sediment was thereafter re-suspended in 2ml of sterile distilled water by 0.5ml transfer pipette. At the end, 0.2 ml of sediment was inoculated onto solid media, Lowenstein Jensen media as per standard protocols [29]. The growth of Mycobacterium tuberculosis bacteria was checked every 7 days up to 8 weeks. For microscopy, a drop of sediment prepared for culture was used for fluorescent acid-fast smear microscopy following the standard procedure [29].

**Data analysis**: Clinical case definition categories for TB in children considered the standardised case definition recently published [30] and were based on clinical screening, X-ray and microbiological investigations. Children were categorized as follows: bacteriologically confirmed TB, unconfirmed TB and unlikely TB (Table 1). Categorical data were interpreted through frequency table with median and interquartile range (IQR) for continuous data. Chi square test or Fisher Exact test was performed to compare the proportion of the outcomes between the groups and 95% confidence intervals (CIs) were calculated for the proportion of an outcome using the binomial exact method. The diagnostic performance of Xpert MTB/RIF assay was compared with the culture method as the primary reference standard. All analyses were conducted using Stata statistical software version 13.1 for Windows [31].

**Ethical approval**: The Senate Research Committee of the University of the Western Cape and the Ethic Review Board of the University of Rwanda, College of Medicine and Health Sciences approved the study protocol. Permission was obtained from Rwanda NTP to collect data from the eligible PHCs.

## Results

**Figure 1:**
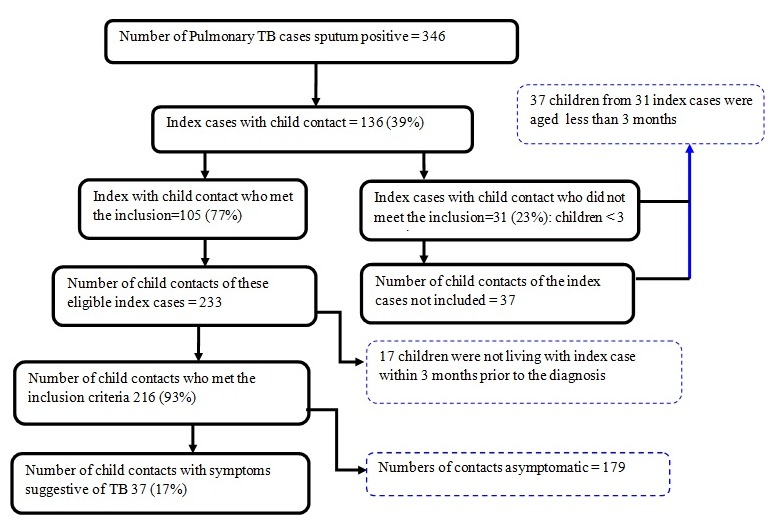
Flow of child contacts recruitment

Figure 1 outlines the study flow chart. There were 346 cases of sputum smear-positive PTB diagnosed and treated in Kigali during the study period from 1st August 2015 and 29th February 2016. Of these 346 index cases, 136 (39%) had at least one child contact and of these 136 index cases, 105 (77%) had a child contact that met the inclusion criteria. The other 31 (23%) index cases with a child contact did not meet inclusion criteria as the child was born after the diagnosis of the index case. Among the 233 child contacts of the 105 eligible index cases, 216 (93%) children met the inclusion criteria of child contacts. The other 17 (17%) were excluded, because they were not living with the index case within the 3 months prior to the diagnosis of the index case. Among these 216 child contacts, 37 (17%) children (derived from 28 index cases) had symptoms suggestive of TB and/or CXR “consistent with active tuberculosis” at the time of screening. Table 2 and Table 3 show the demographic characteristics of the eligible index cases and child contacts, respectively. The results reveal that median age of index cases was 35 years (IQR: 18-65); HIV test was done for 95 (90%) index cases and HIV prevalence was 27%. The findings show that 71 (68%) of all index cases had not yet completed TB treatment. The median age for symptomatic child contacts was 4 years (IQR: 2-13). Among those 37 children, 59% were under five years old, 54% were female, HIV test was done for 31(84%) of them, 3% were HIV positive, and 97% had the evidence of BCG vaccination recorded. IPT had previously been commenced in 84 (89%) of 94 young child contacts without active TB at the time of evaluation. Data of uptake and adherence to IPT will be presented separately once follow-up of the cohort is complete. The majority of child contacts selected in the study were asymptomatic at the time of screening: 179/216 or 83% (95% CI, 77%-87%). All symptomatic child contacts (100%) were exposed to air pollution (tobacco smoke or burning wood) and the majority (64%) had their parents as index cases with 81% in contact with index cases for more than 8 hours per day. In addition, the majorities (81%) of these children were living in the households with more than two people and 78% of those households had just one bedroom. Among the 37 symptomatic child contacts, 92% had at least one symptom suggestive of TB (Table 1) and 10.8% had a CXR “consistent with active tuberculosis”. The most commonly reported symptoms were cough (65%), fever (24%), moderate malnutrition (19%) and enlarged cervical, axillary or inguinal lymph nodes (5%). The CXR was normal in 212 (98%) of all 216 children, whereas 33 (89%) of 37 symptomatic child contacts had a normal CXR. All four abnormal CXRs were reported as “air space opacification”. No asymptomatic child had an abnormal CXR. Of the 37 symptomatic child contacts, 33 ( 89%: 95% CI 73-96) were classified as unlikely TB children and 4 (10.8%: 95% CI 3.9-26.4) had a clinical diagnosis of TB. This represented 1.8% (95% CI, 0.06-0.4) of TB cases among all 216 child contacts. All clinically diagnosed TB cases had at least one symptom suggestive of TB and a CXR consistent with active TB. All these children, who were ≥ 5 years of age, were initiated on TB treatment for six months according to the national guidelines [28] and all completed the TB treatment. None of the symptomatic contacts was bacteriologically confirmed by smear, Xpert MTB/RIF assay or culture on two GL samples.

**Table 2:** Characteristics of the index cases of child contacts

Characteristics	All index cases (n=105) No (%)	Index cases with symptomatic Children (n=28), No (%)
Age median (IQR)	35 (18-65)	33 (19-65)
**Age group in years**		
Female	51 (49)	13 (46)
**Residence of children**		
Nyarugenge	23 (22)	7 (25)
Kicukiro	27 (26)	8 (29)
Gasabo	55 (52)	15 (46)
**Type of health facility**		
Public	70 (67)	16 (57)
Faith based	35 (33)	12 (43)
**Sputum smear**		
Scanty	8 (7.6)	4 (14)
Positive 1	22 (20)	5 (18)
Positive 2	15 (14)	6 (21)
Positive 3	32 (30.4)	9 (32)
Positive 4	28 (27)	4 (14)
Not completed TB treatment	71 (68)	17 (61)
Index case tested for HIV	95 (90)	27 (96)
HIV positive	28 (27)**	6 (22)

Abbreviations: IQR: interquartile range; HIV: human immunodeficiency virus;

** only 95 persons were tested and this number is the denominator.

**Table 3:** Characteristics of child contacts

Characteristics	All child contacts (n=216) No (%)	Symptomatic child contacts (n=37) No (%)
**Age median (IQR)**	6 (2-13)	4 (2-13)
**Age group in years**		
< 5 years	94 (44)	23 (62)
≥ 5 years	122 (56)	15 (41)
**Sex child contact**		
Female	104 (49)	20 (54)
**Sputum smear of index cases**		
Scanty	23 (11)	8 (22)
Positive 1	36 (17)	6 (16)
Positive 2	33 (15)	7 (19)
Positive 3	58 (26)	10 (27)
**BCG scar**	196 (90)	36 (97)
**Nutritional status (Weight for age)**		
Severe malnutrition	0 (0)	0 (0)
Moderate Malnutrition	15 (7)	7 (19)
Normal	201 (93)	30 (81)
HIV positive/tested (%)	5/83 (6)	1 (3)
Receiving IPT /eligible (%)	84/94 (89)	19/23 (82)
**Relationship to Index case**		
Grandchild	15 (7)	5 (14)
Sibling	27 (13)	4 (11)
Child	150 (70)	23 (64)
Others	21 (10)	4 (11)
CXR “consistent with active TB”	4 (1.8)	4 (11)
Spends ≥ 8 hours per day in the same room as the index case	144 (67)	30 (81)
Shares a bed with the index case	150 (69)	15 (41)
Sleeps in the same room as the index case	77 (36)	16 (44)
**Number of people living in the house at the time of the diagnosis**		
One person	36 (17)	7 (19)
Two persons or more	180 (83)	30 (819)
**Number of bedrooms in the house at the time of interview**		
One bedroom	181 (84)	29 (78)
Two bedrooms	27 (13 )	8 (22)
Three bedrooms	7 ( 3)	0 (0)
Indoor air pollution exposure (to tobacco or biomass cooking)	159 (74)	37 (100)

Abbreviations: IQR: interquartile range; BCG: Bacille calmette guerin; IPT: isoniazid preventive therapy

## Discussion

No child contacts were detected with bacteriologically confirmed TB, including those who were symptomatic at the time of screening. Only four (10.8%) children of all symptomatic child contacts were treated for TB based on clinical diagnosis. The very low overall yield (1.8%) of children diagnosed with TB in our study following contact screening is in sharp contrast to the high yield recently reported from Uganda where 10% of 761 contacts were diagnosed with TB of whom 71% were bacteriologically confirmed [32]. A study conducted in Indonesia among 269 child contacts using two separate samples obtained by induced sputum that also included Xpert MTB/RIF assay for M. tuberculosis diagnosed TB in 8% of 269 child contacts, but as in our study, none was bacteriologically confirmed [24]. Contrasting findings have been reported in adult household contacts in Ethiopia [33] where the Xpert MTB/RIF assay in sputum yielded a high percentage of cases (9/14 or 64%) but numbers were small. A possible explanation for a low yield from Xpert MTB/RIF assay is that contact screening may select children with early disease as hospital based studies of children with presumptive TB have had much higher yields [18,23,34-36]. It has been demonstrated that the detection limit of Xpert MTB/RIF assay is low, showing only 131 colony forming unit (CFU) [95% CI: 106-176]/ml of specimen [20,33,37]. Our study also shows that none of the under-five years old child contacts had TB at the time of screening, despite being known to be an “at-risk” group with a high yield of active TB (around 10%) at the time of screening [8,12,13]. This is likely because a large proportion (89%) of child contacts ≤ 5 years were already on IPT at the time of screening. In the studies in Uganda and Indonesia, only 1.5% (7/490) and 0% (0/99), respectively, of eligible child contacts who started on IPT developed active TB [24,32]. Further, there was also a time delay between diagnosis of the index case and contact screening of up to 5 months. There was a low yield from Xpert MTB/RIF assay in sputum collected using GL technique in this study from two sputum samples, which suggests the need to evaluate resource implications and cost-benefit of the policy that recommends Xpert assay for children with presumptive TB who are household contacts [24]. Our study has a number of major limitations. The absence of any confirmed TB cases prevented us from making conclusive remarks about the performance of Xpert MTB/RIF assay as a diagnostic tool in child contacts in sputum using GL technique besides and there was no comparison with other collection methods. Moreover, the small number of TB cases observed could lead to the reduction of the power to detect small differences in the yield between Xpert MTB/RIF assay and clinical diagnosis, microscopy and solid culture.

## Conclusion

The use of Xpert MTB/RIF assay did not contribute to bacteriological confirmation of tuberculosis in child contacts in this study in Rwanda in a setting where there was a high uptake of preventive therapy among eligible child contacts. The low sensitivity of Xpert MTB/RIF assay in contacts may also suggest likely reflection of paucibacillary disease because of early case detection.

### What is known about this topic

- Performance of the Xpert MTB/RIF assay in inpatient and outpatient children (passive case detection);
- Performance of the Xpert MTB/RIF assay in Induce sputum in symptomatic contacts children.

### What this study adds

- Performance of the Xpert MTB/RIF assay in the context of contact screening in contacts children already on IPT;
- Performance of the Xpert MTB/RIF assay in sputum collected by GL in symptomatic contacts children (early case detection).

## Competing interests

The authors declare no competing interests.
